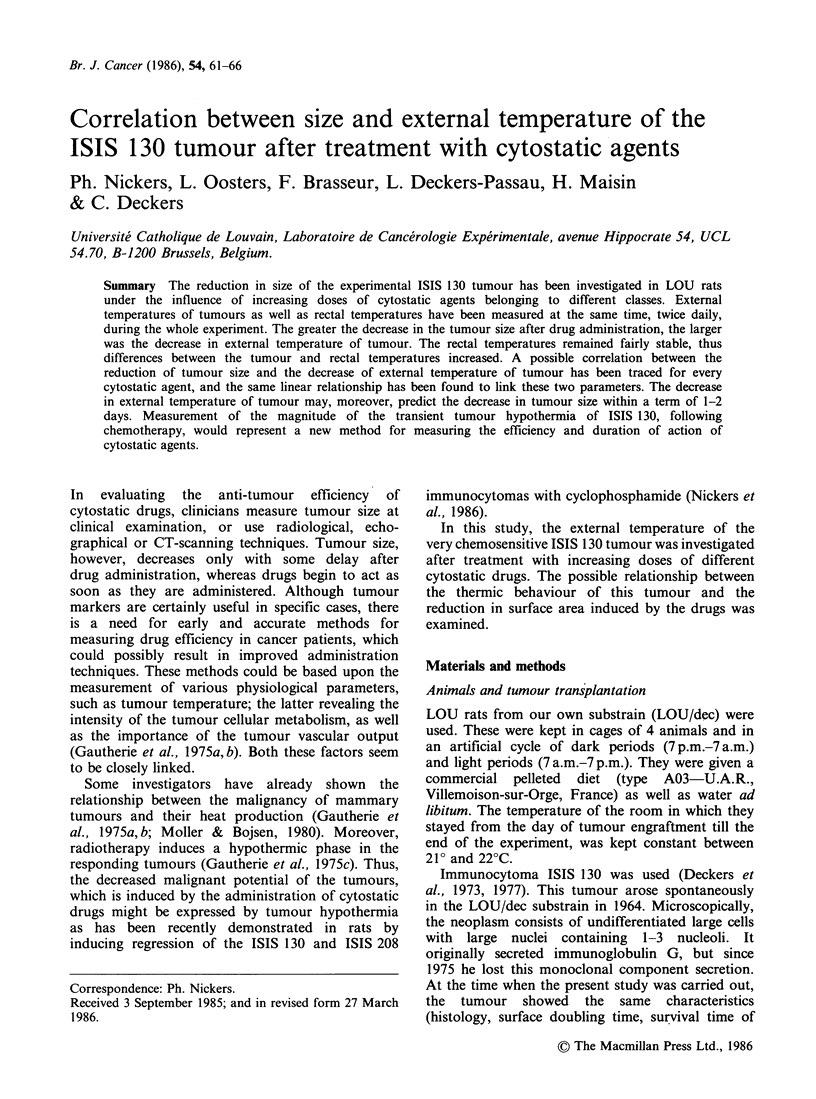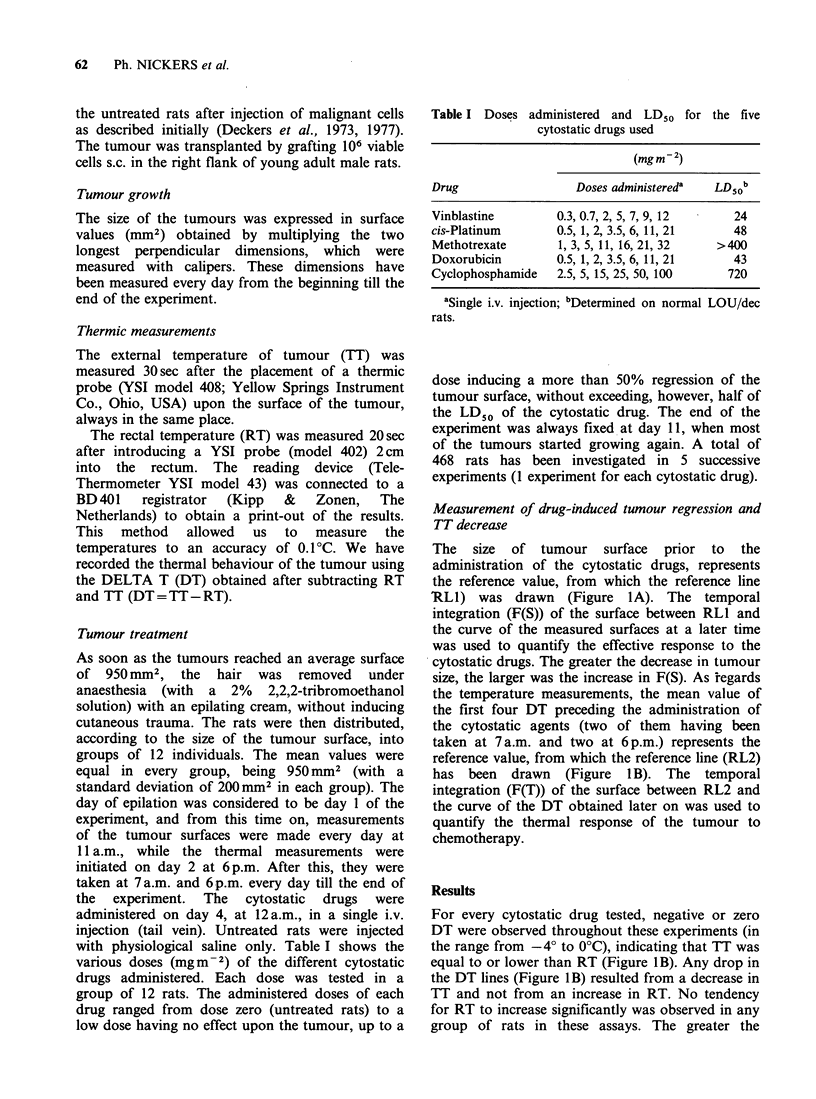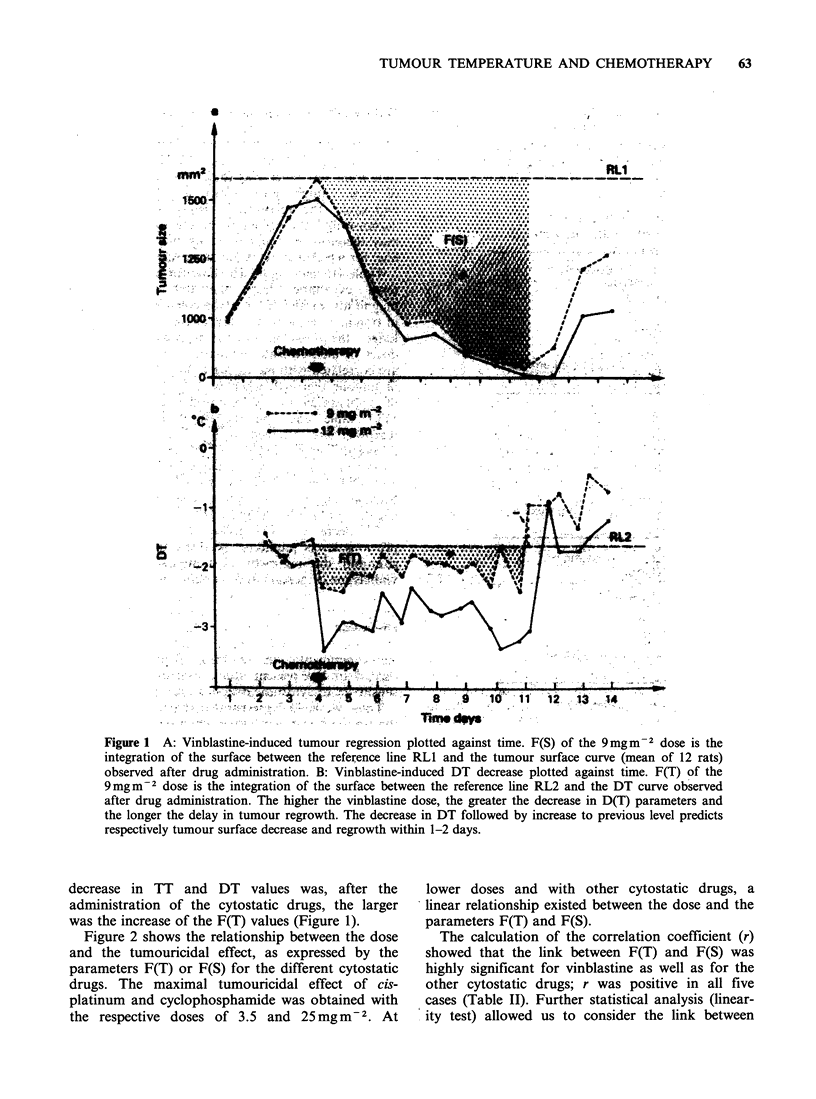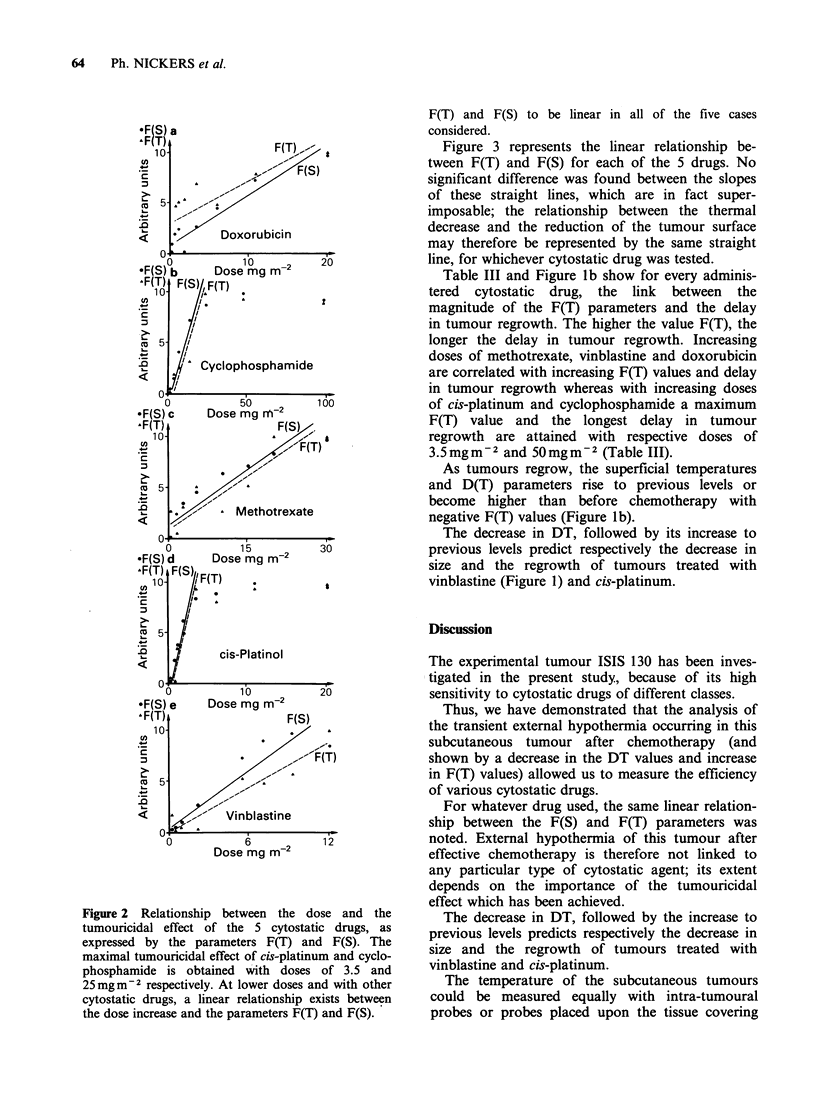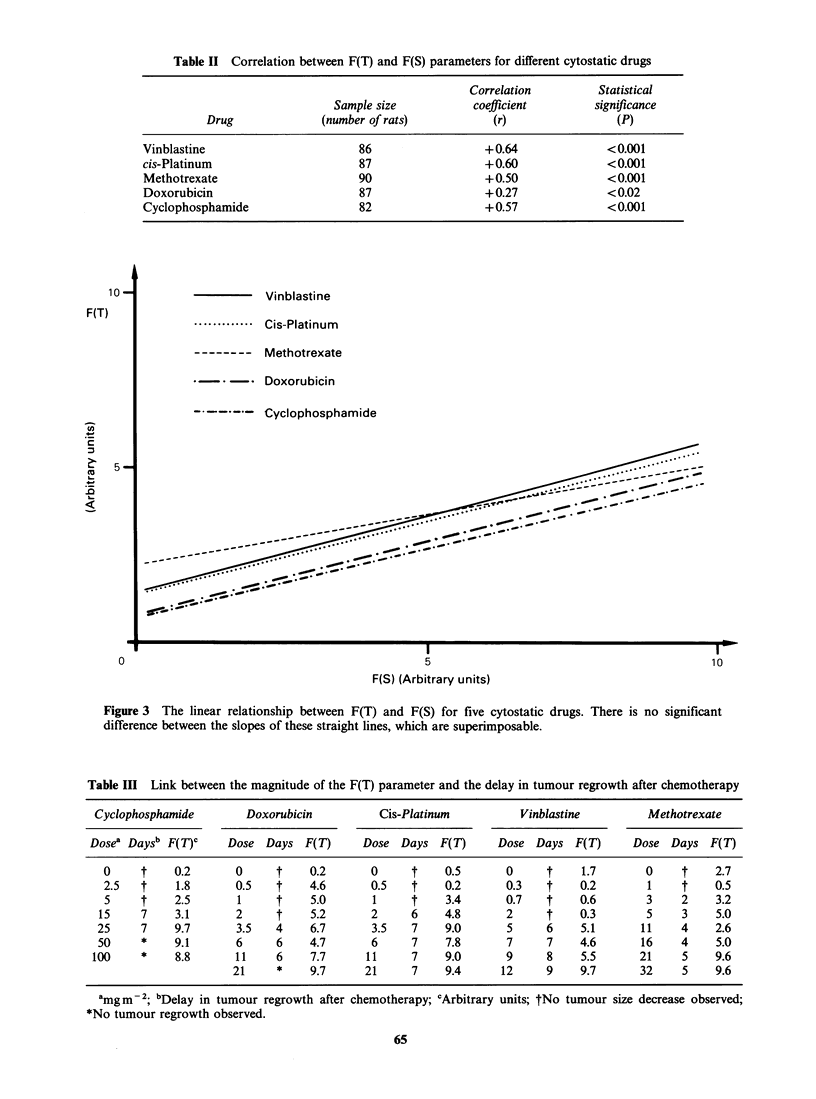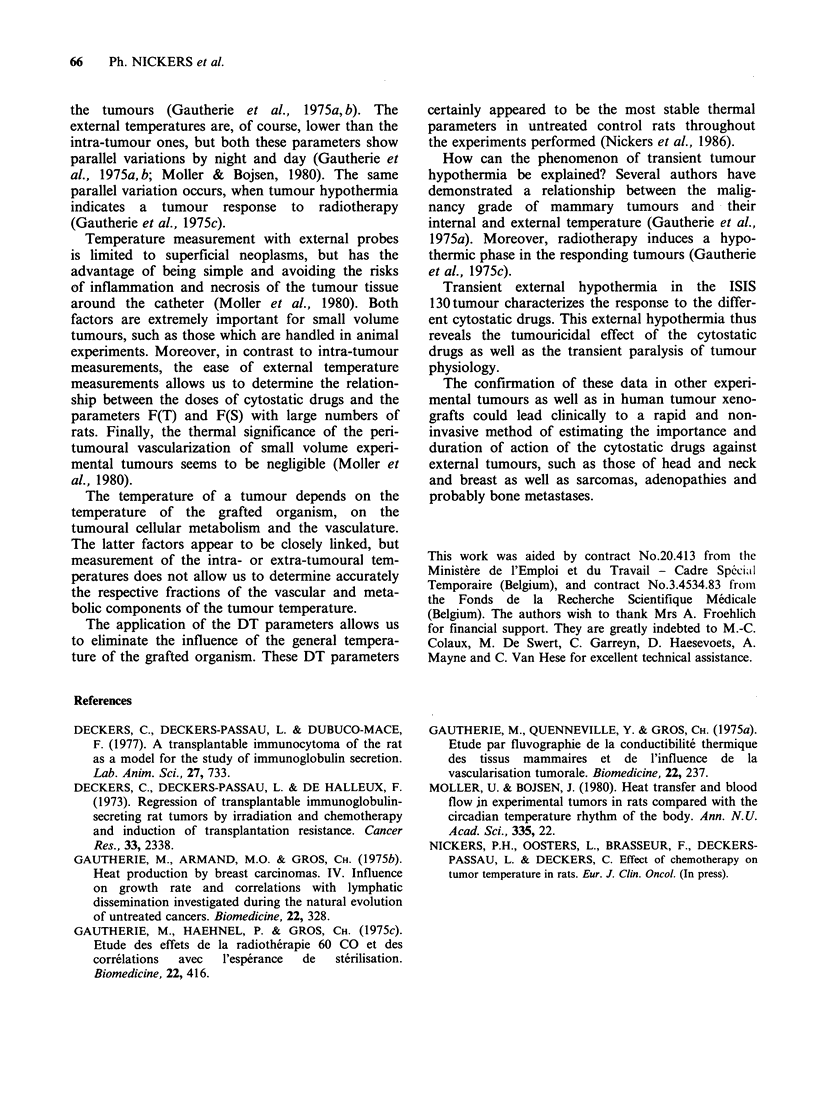# Correlation between size and external temperature of the ISIS 130 tumour after treatment with cytostatic agents.

**DOI:** 10.1038/bjc.1986.152

**Published:** 1986-07

**Authors:** P. Nickers, L. Oosters, F. Brasseur, L. Deckers-Passau, H. Maisin, C. Deckers

## Abstract

The reduction in size of the experimental ISIS 130 tumour has been investigated in LOU rats under the influence of increasing doses of cytostatic agents belonging to different classes. External temperatures of tumours as well as rectal temperatures have been measured at the same time, twice daily, during the whole experiment. The greater the decrease in the tumour size after drug administration, the larger was the decrease in external temperature of tumour. The rectal temperatures remained fairly stable, thus differences between the tumour and rectal temperatures increased. A possible correlation between the reduction of tumour size and the decrease of external temperature of tumour has been traced for every cytostatic agent, and the same linear relationship has been found to link these two parameters. The decrease in external temperature of tumour may, moreover, predict the decrease in tumour size within a term of 1-2 days. Measurement of the magnitude of the transient tumour hypothermia of ISIS 130, following chemotherapy, would represent a new method for measuring the efficiency and duration of action of cytostatic agents.


*Br. J. Cancer* (1986), **54**, 61–66

### Correlation between size and external temperature of the ISIS 130 tumour after treatment with cytostatic agents

Ph. Nickers, L. Oosters, F. Brasseur, L. Deckers-Passau, H. Maisin & C. Deckers

*Université Catholique de Louvain, Laboratoire de Cancérologie Expérimentale, avenue Hippocrate 54, UCL 54.70, B-1200 Brussels, Belgium.*

**Summary** The reduction in size of the experimental ISIS 130 tumour has been investigated in LOU rats under the influence of increasing doses of cytostatic agents belonging to different classes. External temperatures of tumours as well as rectal temperatures have been measured at the same time, twice daily, during the whole experiment. The greater the decrease in the tumour size after drug administration, the larger was the decrease in external temperature of tumour. The rectal temperatures remained fairly stable, thus differences between the tumour and rectal temperatures increased. A possible correlation between the reduction of tumour size and the decrease of external temperature of tumour has been traced for every cytostatic agent, and the same linear relationship has been found to link these two parameters. The decrease in external temperature of tumour may, moreover, predict the decrease in tumour size within a term of 1–2 days. Measurement of the magnitude of the transient tumour hypothermia of ISIS 130, following chemotherapy, would represent a new method for measuring the efficiency and duration of action of cytostatic agents.

In evaluating the anti-tumour efficiency of cytostatic drugs, clinicians measure tumour size at clinical examination, or use radiological, echographical or CT-scanning techniques. Tumour size, however, decreases only with some delay after drug administration, whereas drugs begin to act as soon as they are administered. Although tumour markers are certainly useful in specific cases, there is a need for early and accurate methods for measuring drug efficiency in cancer patients, which could possibly result in improved administration techniques. These methods could be based upon the measurement of various physiological parameters, such as tumour temperature; the latter revealing the intensity of the tumour cellular metabolism, as well as the importance of the tumour vascular output (Gautherie *et al.*, 1975*a*, *b*). Both these factors seem to be closely linked.

Some investigators have already shown the relationship between the malignancy of mammary tumours and their heat production (Gautherie *et al.*, 1975*a*, *b*; Moller & Bojsen, 1980). Moreover, radiotherapy induces a hypothermic phase in the responding tumours (Gautherie *et al.*, 1975*c*). Thus, the decreased malignant potential of the tumours, which is induced by the administration of cytostatic drugs might be expressed by tumour hypothermia as has been recently demonstrated in rats by inducing regression of the ISIS 130 and ISIS 208 immunocytomas with cyclophosphamide (Nickers *et al.*, 1986).

In this study, the external temperature of the very chemosensitive ISIS 130 tumour was investigated after treatment with increasing doses of different cytostatic drugs. The possible relationship between the thermic behaviour of this tumour and the reduction in surface area induced by the drugs was examined.

### Materials and methods

### *Animals and tumour transplantation*

LOU rats from our own substrain (LOU/dec) were used. These were kept in cages of 4 animals and in an artificial cycle of dark periods (7 p.m.–7 a.m.) and light periods (7 a.m.–7 p.m.). They were given a commercial pelleted diet (type A03—U.A.R., Villemoison-sur-Orge, France) as well as water *ad libitum*. The temperature of the room in which they stayed from the day of tumour engraftment till the end of the experiment, was kept constant between 21° and 22°C.

Immunocytoma ISIS 130 was used (Deckers *et al.*, 1973, 1977). This tumour arose spontaneously in the LOU/dec substrain in 1964. Microscopically, the neoplasm consists of undifferentiated large cells with large nuclei containing 1–3 nucleoli. It originally secreted immunoglobulin G, but since 1975 he lost this monoclonal component secretion. At the time when the present study was carried out, the tumour showed the same characteristics (histology, surface doubling time, survival time of

Correspondence: Ph. Nickers.
Received 3 September 1985; and in revised form 27 March 1986.

© The Macmillan Press Ltd., 1986

the untreated rats after injection of malignant cells as described initially (Deckers *et al.*, 1973, 1977). The tumour was transplanted by grafting $10^6$ viable cells s.c. in the right flank of young adult male rats.

*Tumour growth*

The size of the tumours was expressed in surface values ($mm^2$) obtained by multiplying the two longest perpendicular dimensions, which were measured with calipers. These dimensions have been measured every day from the beginning till the end of the experiment.

*Thermic measurements*

The external temperature of tumour (TT) was measured 30 sec after the placement of a thermic probe (YSI model 408; Yellow Springs Instrument Co., Ohio, USA) upon the surface of the tumour, always in the same place.

The rectal temperature (RT) was measured 20 sec after introducing a YSI probe (model 402) 2 cm into the rectum. The reading device (Tele-Thermometer YSI model 43) was connected to a BD 401 registrator (Kipp & Zonen, The Netherlands) to obtain a print-out of the results. This method allowed us to measure the temperatures to an accuracy of 0.1°C. We have recorded the thermal behaviour of the tumour using the DELTA T (DT) obtained after subtracting RT and TT (DT = TT − RT).

*Tumour treatment*

As soon as the tumours reached an average surface of 950 $mm^2$, the hair was removed under anaesthesia (with a 2% 2,2,2-tribromoethanol solution) with an epilating cream, without inducing cutaneous trauma. The rats were then distributed, according to the size of the tumour surface, into groups of 12 individuals. The mean values were equal in every group, being 950 $mm^2$ (with a standard deviation of 200 $mm^2$ in each group). The day of epilation was considered to be day 1 of the experiment, and from this time on, measurements of the tumour surfaces were made every day at 11 a.m., while the thermal measurements were initiated on day 2 at 6 p.m. After this, they were taken at 7 a.m. and 6 p.m. every day till the end of the experiment. The cytostatic drugs were administered on day 4, at 12 a.m., in a single i.v. injection (tail vein). Untreated rats were injected with physiological saline only. Table I shows the various doses ($mg\,m^{-2}$) of the different cytostatic drugs administered. Each dose was tested in a group of 12 rats. The administered doses of each drug ranged from dose zero (untreated rats) to a low dose having no effect upon the tumour, up to a dose inducing a more than 50% regression of the tumour surface, without exceeding, however, half of the $LD_{50}$ of the cytostatic drug. The end of the experiment was always fixed at day 11, when most of the tumours started growing again. A total of 468 rats has been investigated in 5 successive experiments (1 experiment for each cytostatic drug).

**Table I** Doses administered and $LD_{50}$ for the five cytostatic drugs used

| | ($mg\,m^{-2}$) | |
|---|---|---|
| *Drug* | *Doses administered*[a] | $LD_{50}$[b] |
| Vinblastine | 0.3, 0.7, 2, 5, 7, 9, 12 | 24 |
| *cis*-Platinum | 0.5, 1, 2, 3.5, 6, 11, 21 | 48 |
| Methotrexate | 1, 3, 5, 11, 16, 21, 32 | >400 |
| Doxorubicin | 0.5, 1, 2, 3.5, 6, 11, 21 | 43 |
| Cyclophosphamide | 2.5, 5, 15, 25, 50, 100 | 720 |

[a]Single i.v. injection; [b]Determined on normal LOU/dec rats.

*Measurement of drug-induced tumour regression and TT decrease*

The size of tumour surface prior to the administration of the cytostatic drugs, represents the reference value, from which the reference line RL1) was drawn (Figure 1A). The temporal integration (F(S)) of the surface between RL1 and the curve of the measured surfaces at a later time was used to quantify the effective response to the cytostatic drugs. The greater the decrease in tumour size, the larger was the increase in F(S). As regards the temperature measurements, the mean value of the first four DT preceding the administration of the cytostatic agents (two of them having been taken at 7 a.m. and two at 6 p.m.) represents the reference value, from which the reference line (RL2) has been drawn (Figure 1B). The temporal integration (F(T)) of the surface between RL2 and the curve of the DT obtained later on was used to quantify the thermal response of the tumour to chemotherapy.

### Results

For every cytostatic drug tested, negative or zero DT were observed throughout these experiments (in the range from −4° to 0°C), indicating that TT was equal to or lower than RT (Figure 1B). Any drop in the DT lines (Figure 1B) resulted from a decrease in TT and not from an increase in RT. No tendency for RT to increase significantly was observed in any group of rats in these assays. The greater the

**Figure 1** A: Vinblastine-induced tumour regression plotted against time. F(S) of the $9\,mg\,m^{-2}$ dose is the integration of the surface between the reference line RL1 and the tumour surface curve (mean of 12 rats) observed after drug administration. B: Vinblastine-induced DT decrease plotted against time. F(T) of the $9\,mg\,m^{-2}$ dose is the integration of the surface between the reference line RL2 and the DT curve observed after drug administration. The higher the vinblastine dose, the greater the decrease in D(T) parameters and the longer the delay in tumour regrowth. The decrease in DT followed by increase to previous level predicts respectively tumour surface decrease and regrowth within 1–2 days.

decrease in TT and DT values was, after the administration of the cytostatic drugs, the larger was the increase of the F(T) values (Figure 1).

Figure 2 shows the relationship between the dose and the tumouricidal effect, as expressed by the parameters F(T) or F(S) for the different cytostatic drugs. The maximal tumouricidal effect of *cis*-platinum and cyclophosphamide was obtained with the respective doses of 3.5 and $25\,mg\,m^{-2}$. At lower doses and with other cytostatic drugs, a linear relationship existed between the dose and the parameters F(T) and F(S).

The calculation of the correlation coefficient (*r*) showed that the link between F(T) and F(S) was highly significant for vinblastine as well as for the other cytostatic drugs; *r* was positive in all five cases (Table II). Further statistical analysis (linearity test) allowed us to consider the link between

**Figure 2** Relationship between the dose and the tumouricidal effect of the 5 cytostatic drugs, as expressed by the parameters F(T) and F(S). The maximal tumouricidal effect of *cis*-platinum and cyclophosphamide is obtained with doses of 3.5 and 25 mg m$^{-2}$ respectively. At lower doses and with other cytostatic drugs, a linear relationship exists between the dose increase and the parameters F(T) and F(S).

F(T) and F(S) to be linear in all of the five cases considered.

Figure 3 represents the linear relationship between F(T) and F(S) for each of the 5 drugs. No significant difference was found between the slopes of these straight lines, which are in fact superimposable; the relationship between the thermal decrease and the reduction of the tumour surface may therefore be represented by the same straight line, for whichever cytostatic drug was tested.

Table III and Figure 1b show for every administered cytostatic drug, the link between the magnitude of the F(T) parameters and the delay in tumour regrowth. The higher the value F(T), the longer the delay in tumour regrowth. Increasing doses of methotrexate, vinblastine and doxorubicin are correlated with increasing F(T) values and delay in tumour regrowth whereas with increasing doses of *cis*-platinum and cyclophosphamide a maximum F(T) value and the longest delay in tumour regrowth are attained with respective doses of 3.5 mg m$^{-2}$ and 50 mg m$^{-2}$ (Table III).

As tumours regrow, the superficial temperatures and D(T) parameters rise to previous levels or become higher than before chemotherapy with negative F(T) values (Figure 1b).

The decrease in DT, followed by its increase to previous levels predict respectively the decrease in size and the regrowth of tumours treated with vinblastine (Figure 1) and *cis*-platinum.

### Discussion

The experimental tumour ISIS 130 has been investigated in the present study., because of its high sensitivity to cytostatic drugs of different classes.

Thus, we have demonstrated that the analysis of the transient external hypothermia occurring in this subcutaneous tumour after chemotherapy (and shown by a decrease in the DT values and increase in F(T) values) allowed us to measure the efficiency of various cytostatic drugs.

For whatever drug used, the same linear relationship between the F(S) and F(T) parameters was noted. External hypothermia of this tumour after effective chemotherapy is therefore not linked to any particular type of cytostatic agent; its extent depends on the importance of the tumouricidal effect which has been achieved.

The decrease in DT, followed by the increase to previous levels predicts respectively the decrease in size and the regrowth of tumours treated with vinblastine and *cis*-platinum.

The temperature of the subcutaneous tumours could be measured equally with intra-tumoural probes or probes placed upon the tissue covering

**Table II** Correlation between F(T) and F(S) parameters for different cytostatic drugs

| *Drug* | *Sample size (number of rats)* | *Correlation coefficient (r)* | *Statistical significance (P)* |
|---|---|---|---|
| Vinblastine | 86 | +0.64 | <0.001 |
| *cis*-Platinum | 87 | +0.60 | <0.001 |
| Methotrexate | 90 | +0.50 | <0.001 |
| Doxorubicin | 87 | +0.27 | <0.02 |
| Cyclophosphamide | 82 | +0.57 | <0.001 |

**Figure 3** The linear relationship between F(T) and F(S) for five cytostatic drugs. There is no significant difference between the slopes of these straight lines, which are superimposable.

**Table III** Link between the magnitude of the F(T) parameter and the delay in tumour regrowth after chemotherapy

| *Cyclophosphamide* | | | *Doxorubicin* | | | Cis-*Platinum* | | | *Vinblastine* | | | *Methotrexate* | | |
|---|---|---|---|---|---|---|---|---|---|---|---|---|---|---|
| *Dose*[a] | *Days*[b] | *F(T)*[c] | *Dose* | *Days* | *F(T)* | *Dose* | *Days* | *F(T)* | *Dose* | *Days* | *F(T)* | *Dose* | *Days* | *F(T)* |
| 0 | † | 0.2 | 0 | † | 0.2 | 0 | † | 0.5 | 0 | † | 1.7 | 0 | † | 2.7 |
| 2.5 | † | 1.8 | 0.5 | † | 4.6 | 0.5 | † | 0.2 | 0.3 | † | 0.2 | 1 | † | 0.5 |
| 5 | † | 2.5 | 1 | † | 5.0 | 1 | † | 3.4 | 0.7 | † | 0.6 | 3 | 2 | 3.2 |
| 15 | 7 | 3.1 | 2 | † | 5.2 | 2 | 6 | 4.8 | 2 | † | 0.3 | 5 | 3 | 5.0 |
| 25 | 7 | 9.7 | 3.5 | 4 | 6.7 | 3.5 | 7 | 9.0 | 5 | 6 | 5.1 | 11 | 4 | 2.6 |
| 50 | * | 9.1 | 6 | 6 | 4.7 | 6 | 7 | 7.8 | 7 | 7 | 4.6 | 16 | 4 | 5.0 |
| 100 | * | 8.8 | 11 | 6 | 7.7 | 11 | 7 | 9.0 | 9 | 8 | 5.5 | 21 | 5 | 9.6 |
| | | | 21 | * | 9.7 | 21 | 7 | 9.4 | 12 | 9 | 9.7 | 32 | 5 | 9.6 |

[a]$mg\,m^{-2}$; [b]Delay in tumour regrowth after chemotherapy; [c]Arbitrary units; †No tumour size decrease observed; *No tumour regrowth observed.

the tumours (Gautherie *et al.*, 1975*a,b*). The external temperatures are, of course, lower than the intra-tumour ones, but both these parameters show parallel variations by night and day (Gautherie *et al.*, 1975*a,b*; Moller & Bojsen, 1980). The same parallel variation occurs, when tumour hypothermia indicates a tumour response to radiotherapy (Gautherie *et al.*, 1975*c*).

Temperature measurement with external probes is limited to superficial neoplasms, but has the advantage of being simple and avoiding the risks of inflammation and necrosis of the tumour tissue around the catheter (Moller *et al.*, 1980). Both factors are extremely important for small volume tumours, such as those which are handled in animal experiments. Moreover, in contrast to intra-tumour measurements, the ease of external temperature measurements allows us to determine the relationship between the doses of cytostatic drugs and the parameters F(T) and F(S) with large numbers of rats. Finally, the thermal significance of the peri-tumoural vascularization of small volume experimental tumours seems to be negligible (Moller *et al.*, 1980).

The temperature of a tumour depends on the temperature of the grafted organism, on the tumoural cellular metabolism and the vasculature. The latter factors appear to be closely linked, but measurement of the intra- or extra-tumoural temperatures does not allow us to determine accurately the respective fractions of the vascular and metabolic components of the tumour temperature.

The application of the DT parameters allows us to eliminate the influence of the general temperature of the grafted organism. These DT parameters certainly appeared to be the most stable thermal parameters in untreated control rats throughout the experiments performed (Nickers *et al.*, 1986).

How can the phenomenon of transient tumour hypothermia be explained? Several authors have demonstrated a relationship between the malignancy grade of mammary tumours and their internal and external temperature (Gautherie *et al.*, 1975*a*). Moreover, radiotherapy induces a hypothermic phase in the responding tumours (Gautherie *et al.*, 1975*c*).

Transient external hypothermia in the ISIS 130 tumour characterizes the response to the different cytostatic drugs. This external hypothermia thus reveals the tumouricidal effect of the cytostatic drugs as well as the transient paralysis of tumour physiology.

The confirmation of these data in other experimental tumours as well as in human tumour xenografts could lead clinically to a rapid and non-invasive method of estimating the importance and duration of action of the cytostatic drugs against external tumours, such as those of head and neck and breast as well as sarcomas, adenopathies and probably bone metastases.

This work was aided by contract No.20.413 from the Ministère de l'Emploi et du Travail – Cadre Spécial Temporaire (Belgium), and contract No.3.4534.83 from the Fonds de la Recherche Scientifique Médicale (Belgium). The authors wish to thank Mrs A. Froehlich for financial support. They are greatly indebted to M.-C. Colaux, M. De Swert, C. Garreyn, D. Haesevoets, A. Mayne and C. Van Hese for excellent technical assistance.

**References**

DECKERS, C., DECKERS-PASSAU, L. & DUBUCO-MACE, F. (1977). A transplantable immunocytoma of the rat as a model for the study of immunoglobulin secretion. *Lab. Anim. Sci.*, **27,** 733.

DECKERS, C., DECKERS-PASSAU, L. & DE HALLEUX, F. (1973). Regression of transplantable immunoglobulin-secreting rat tumors by irradiation and chemotherapy and induction of transplantation resistance. *Cancer Res.*, **33,** 2338.

GAUTHERIE, M., ARMAND, M.O. & GROS, CH. (1975*b*). Heat production by breast carcinomas. IV. Influence on growth rate and correlations with lymphatic dissemination investigated during the natural evolution of untreated cancers. *Biomedicine*, **22,** 328.

GAUTHERIE, M., HAEHNEL, P. & GROS, CH. (1975*c*). Etude des effets de la radiothérapie 60 CO et des corrélations avec l'espérance de stérilisation. *Biomedicine*, **22,** 416.

GAUTHERIE, M., QUENNEVILLE, Y. & GROS, CH. (1975*a*). Etude par fluvographie de la conductibilité thermique des tissus mammaires et de l'influence de la vascularisation tumorale. *Biomedicine*, **22,** 237.

MOLLER, U. & BOJSEN, J. (1980). Heat transfer and blood flow in experimental tumors in rats compared with the circadian temperature rhythm of the body. *Ann. N.U. Acad. Sci.*, **335,** 22.

NICKERS, P.H., OOSTERS, L., BRASSEUR, F., DECKERS-PASSAU, L. & DECKERS, C. Effect of chemotherapy on tumor temperature in rats. *Eur. J. Clin. Oncol.* (In press).